# Interleukin-35 Attenuates D-Galactosamine/Lipopolysaccharide-Induced Liver Injury via Enhancing Interleukin-10 Production in Kupffer Cells

**DOI:** 10.3389/fphar.2018.00959

**Published:** 2018-08-24

**Authors:** Xing-Feng Zheng, Xiao-Yan Hu, Bing Ma, He Fang, Fang Zhang, Yan-Fei Mao, Feng-Yong Yang, Shi-Chu Xiao, Zhao-Fan Xia

**Affiliations:** ^1^Department of Burn Surgery, Changhai Hospital, The Second Military Medical University, Shanghai, China; ^2^Department of Anesthesiology and Surgical Intensive Care Unit, Xinhua Hospital, Shanghai Jiao Tong University School of Medicine, Shanghai, China; ^3^Intensive Care Unit, The People’s Hospital of Laiwu City, Laiwu, China

**Keywords:** interleukin-35, hepatitis, lipopolysaccharide, Kupffer cell, interleukin-10

## Abstract

Interleukin (IL) -35 is an anti-inflammatory cytokine which exerts various beneficial effects on autoimmune diseases. However, whether IL-35 plays a role in endotoxin induced hepatitis demands clarification. This study aims to reveal the effect and mechanism of IL-35 on endotoxin induced liver injury. Acute hepatic injury was induced by D-galactosamine (D-GalN, 400 mg/kg) and lipopolysaccharide (LPS, 5 μg/kg) administration in mice. IL-35 treatment ameliorated D-GalN/LPS induced liver injury in a dose dependent manner as shown by histological examination, ALT determination and Caspase-3 activity assay. It also reduced production of pro-inflammatory cytokines, tumor necrosis factor (TNF)-α, IL-1β, and IL-6, and increased production of anti-inflammatory cytokines, IL-4, IL-10, and transforming growth factor (TGF)-β. This hepato-protective effect was proved mainly mediated by Kupffer cells (KC) via gadolinium chloride depletion and cell adoptive transfer experiment. In addition, IL-35 emolliated the cytotoxicity of LPS-triggered KCs to hepatocytes, suppressed nitric oxide (NO) and TNF-α production, and elevated IL-10 production in LPS stimulated KCs. Furthermore, IL-35 could not exert hepato-protective effect in IL-10-deficient mice *in vivo* and it could not suppress LPS induced NO and TNF-α production in IL-10-deficient KCs *in vitro*. In conclusion, IL-35 protects endotoxin-induced acute liver injury, which mainly acts thought increasing IL-10 production in KCs. This finding demonstrates a role of IL-35 in anti-infectious immunity and provides a potential therapeutic target in treating fulminant hepatitis.

## Introduction

Fulminant hepatitis is a severe clinical syndrome associated with a high rate of mortality caused by diverse reasons (Mas and Rodes, 1997). Endotoxin plays a major part in the develop of various type of acute fulminant hepatitis (Nolan, 2010). Lipopolysaccharide (LPS) induced liver injury in D-galactosamine (D-GalN) sensitized mice is a well-established animal model widely used in exploring the pathogenesis of fulminant hepatitis (Mignon et al., 1999). In addition, evidence has indicated that Kupffer cells (KCs) were involved in the develop of hepatitis (Yang et al., 2004). Interleukin (IL) -10 was considered to be a critical factor responsible for immune tolerance in hepatitis (Erhardt et al., 2007; Kamimoto et al., 2009; Xu et al., 2014).

Interleukin -35 is a member of the IL-12 family, whose biological effects and clinical significance are growingly recognized (Collison et al., 2010). It is considered that IL-35 is mostly secreted by regulatory T (Treg) cells (Collison et al., 2007) and regulatory B (Breg) cells (Wang et al., 2014). It functions as an immunosuppressive cytokine, involved in arthritis (Niedbala et al., 2007), inflammatory bowel disease (IBD) (Collison et al., 2007), psoriasis (Wang et al., 2018), uveitis (Wang et al., 2014), diabetes (Manzoor et al., 2017), and other autoimmune diseases.

It was also reported that IL-35 was elevated in chronic Hepatitis B patients (Liu et al., 2011; Xiang and Xie, 2015; Zhou et al., 2015). However, whether IL-35 exerts protective effect on endotoxin induced hepatic injury is not clarified. Therefore, the effect of IL-35 on LPS induced liver injury in D-GalN sensitized mice is investigated and whether this effect is mediated by KCs and IL-10 is also verified in this study.

## Materials and Methods

### Animals and Reagents

Adult male C57BL/6 mice were obtained from Sipper BK Experimental Animals, Co. (Shanghai, China). IL-10 knockout mice (C57BL/6 background) were purchased from Jackson Laboratory (Bar Harbor, ME, United States). All mice were housed under SPF facility in a 12:12 light–dark cycle and used for animal experiments at age of 6–8 weeks. All procedures were performed according to the National Institutes of Health Guide for the Care and Use of Laboratory Animals and were approved by the Animal Ethics Committee of the Second Military Medical University. Mouse recombinant IL-35 was purchased from AdipoGen (Liestal, Switzerland). LPS (Escherichia coli, O26:B6), D-GalN, gadolinium chloride (GdCl_3_), 7-AAD, and bovine serum albumin (BSA) were from Sigma (St. Louis, MO, United States). Fluorescein-conjugated mAbs against Annxin V and F4/80 were obtained from BD-PharMingen (San Diego, CA, United States). Sources of the other antibodies and kits are described in the methods below.

### *In vivo* Experiment Design

Acute hepatic injury in mice was established by injection with D-GalN (400 mg/kg body weight) and LPS (5 μg/kg body weight) intraperitoneally as described (Jiang et al., 2005). For dose-response experiment, animals were randomized into four groups (*n* = 10). IL-35 of different concentrations as indicated (1, 10, 100 ug/kg BW) or normal saline as control was administered intraperitoneally 5 min after D-GalN /LPS injection. For cytokine comparing experiment, animals were randomized into two groups (*n* = 10). IL-35 (100 ug/kg BW) or normal saline as control was administered. To evaluate role of IL-10, both IL-10-deficient mice and wild type mice were randomized into two groups (*n* = 8). IL-35 (100 ug/kg) or normal saline as control was administered. For ALT determination and histological observation, animals were sacrificed 24 h after D-GalN/LPS treatment. For caspase-3 activity and cytokine measurement, animals were sacrificed 8 h after D-GalN/LPS treatment. Liver tissues were excised, weighed and processed for histopathology or frozen at -80°C until use for other assays, and blood was drawn for separation of serum.

### Cell Preparations

Hepatocytes and liver KCs were prepared from mice according to the previous method (Zhang et al., 2009). Briefly, the liver cells were dispersed by two-step collagenase perfusion method. The cell suspension was filtered through two layers of nylon mesh and the hepatocytes were pelleted by low-speed centrifugation. KCs were isolated by density gradient centrifugation in the supernatant. Cell viability was assessed by trypan blue exclusion. Cells were resuspended in William’s medium E containing 10% fetal calf serum and cultured at 37°C in a humidified incubator (95% air/5% CO_2_). Further purification of the cells was achieved by attachment to the plastic plates for 2–3 h. Medium was renewed after 3 h and every other day. All cells were cultured for 1–3 days before being used in the experiments. Cytotoxicity of LPS-triggered KCs to hepatocytes was assayed as described. KCs were co-cultured with hepatocytes at the E: T (KC: hepatocytes) ratio of 1:10. The number of live hepatocytes was determined using anti-F4/80-APC Ab, Annexin V-FITC, and 7-AAD. The F4/80 antigen was expressed on mature tissue macrophages widely including KCs, so the F4/80 negative cells in our co-culture system were mostly hepatocytes. Annexin V-PE was used to detect phosphatidylserine (PS) which was exposed on the external membrane of apoptotic cells. Meanwhile, 7-AAD only permeated in the late-stage apoptotic and dead cells because it could be excluded from live and healthy cells. So 7AAD^−^Annexin V^−^ cells were considered as live cells. The cytolysis (%) of hepatocytes in co-culture system was calculated as [1 - (7AAD^−^Annexin V^−^F4/80^−^/F4/80^−^)] × 100. KCs were cultured 3 days before LPS stimulation (100 ng/ml) for 12 h (Zhang et al., 2011).

### Cell Depletion and Cell Adoptive Transfer

For KCs depletion experiment, animals were randomized into four groups (*n* = 8): GdCl_3_ + KCs (IL-35), GdCl_3_ + KCs (PBS), GdCl_3_ + PBS, and PBS + PBS. Mice were intravenously (i.v.) injected of GdCl_3_ (20 mg/kg body weight) 24 h before D-GalN/LPS treatment for KCs depletion groups. For adoptive cell transfer, mice were received portal vein (p.v.) injection of KCs (1 × 10^6^/mouse) 24 h after GdCl_3_ treatment, while the transferred KCs were pretreated with or without IL-35 (10 ng/ml) 1 day prior to injection.

### Histopathological Study

Small pieces of the liver were fixed in 10% buffered formalin and subsequently embedded in paraffin. After hematoxylin and eosin (H&E) staining, tissue slices were examined under the microscope by two independent and blinded researchers to assess the degree of histological features in acute hepatic injury. Three sections of liver from each mouse (ten mice per group) were examined. The histological severity of liver injury was graded using Suzuki’s criteria. In brief, sinusoidal congestion, hepatocyte necrosis, and ballooning degeneration were graded from 0 to 4. No necrosis, congestion or centrilobular ballooning was given a score of 0, whereas severe congestion and ballooning degeneration as well as >60% lobular necrosis was given a value of four.

### Serum Biochemistry

Serum alanine aminotransferase (ALT) was determined using commercial kits by an automatic blood biochemical analyzer (7600-120, Hitachi High-Technologies, Corp., Tokyo, Japan).

### Caspase-3 Activities Determination

Liver Caspase-3 activities were assayed using fluorometric caspase activity measuring kits (Calbiochem, La Jolla, CA, United States) (Catalog No. QIA70) and read in FlexStation^®^ II Microplate Reader (Molecular Devices, Sunnyvale, CA, United States) as described (Neumar et al., 2003). Determination of protein concentration was made on the homogenates with BCA protein assay kit (Pierce, Rockford, IL, United States) (Catalog No. 23225) using BSA as the standard.

### Determination of Cytokines and Nitric Oxide (NO)

Cytokines concentrations were detected by enzyme-linked immunosorbent assay (ELISA) assay using commercial kits from R&D Systems (Minneapolis, MN, United States) according to the manufacturer’s recommendations. The Catalog No. of cytokine detecting kits were as following: mouse TNF-alpha DuoSet ELISA DY410-05, mouse IL-10 DuoSet ELISA DY417-05, mouse TGF-beta 1 DuoSet ELISA DY1679-05, mouse IL-1 beta/IL-1F2 DuoSet ELISA DY401-05, mouse IL-6 DuoSet ELISA DY406-05, mouse IL-4 DuoSet ELISA DY404-05. Most of the NO in culture system was oxidized to nitrite and nitrate and the nitrate was eventually conversed to nitrite. So, the presence of NO was measured by assay for nitrite, a stable product of NO in solution, using Griess Reagent Kit (Catalog No. G7921) for nitrite quantitation (Invitrogen, Eugene, OR, United States).

### Statistical Analysis

All data are expressed as the mean ± SEM. Results were analyzed by computerized statistical packages (SPSS). Each mean value was compared by one-way analysis of variance and Student-Newman-Keuls for multiple comparisons as the *post hoc* test. *P*-values less than 0.05 were considered statistically significant.

## Results

### IL-35 Ameliorated D-GalN/LPS Induced Hepatic Injury

**FIGURE 1:**
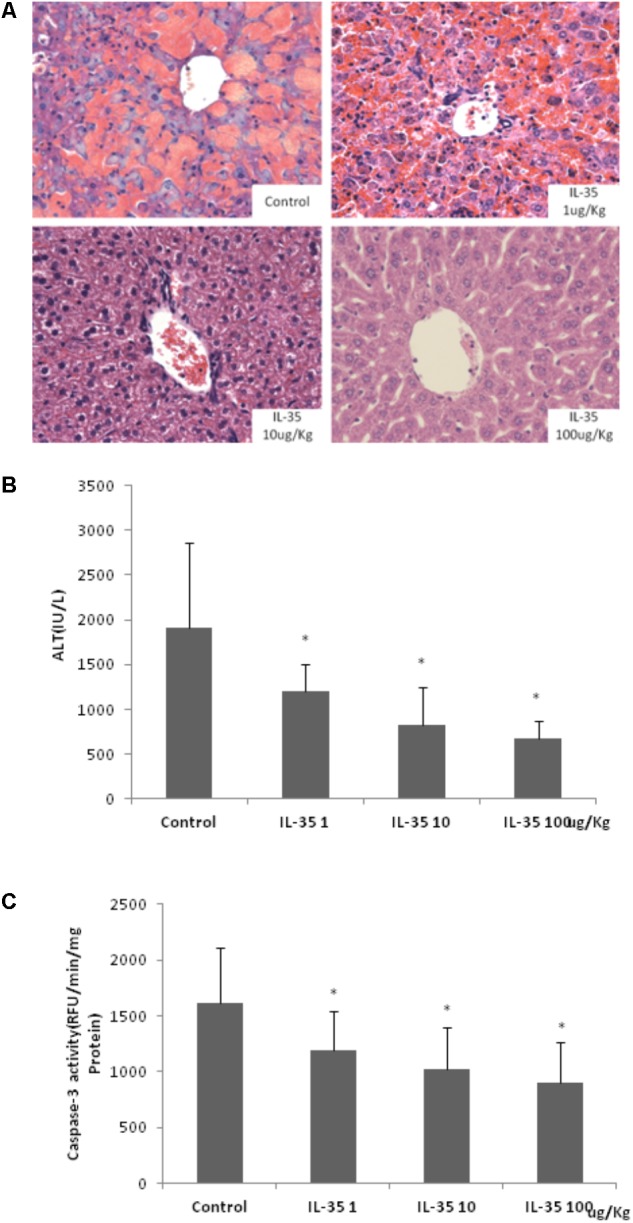
IL-35 suppresses endotoxin-induced hepatitis in a dose-dependent manner. **(A)** Histological analysis of liver tissue in C57BL/6 mice (*n* = 10) after D-GalN/LPS challenge under different doses of IL-35. **(B)** Serum ALT determination in C57BL/6 mice (*n* = 10) after D-GalN/LPS challenge under different doses of IL-35. **(C)** Caspase-3 analysis of liver tissue in C57BL/6 mice (*n* = 10) after D-GalN/LPS challenge under different doses of IL-35. ^∗^*P* < 0.01 vs. control group. Data are representative of three independent experiments.

Aminotransferase level in healthy mice was 40.20 ± 2.48 IU/L (not shown in Figure). D-GalN/LPS induced an obvious acute fulminant hepatitis in mice. IL-35 administration suppressed D-GalN/LPS induced liver injury in a dose dependent manner, which was observed from the histological assay of HE staining (**Figure 1A**) and serum ALT determination (**Figure 1B**). Livers from GalN/LPS-treated mice showed extensive injury, specifically necrosis, inflammation and hemorrhage changes around the central veins. On the other hand, IL-35 treatment obviously relieved these pathological changes. It also emolliated hepatocytes apoptosis in a dose dependent manner, which demonstrated by Caspase-3 activity (**Figure 1C**).

**FIGURE 2:**
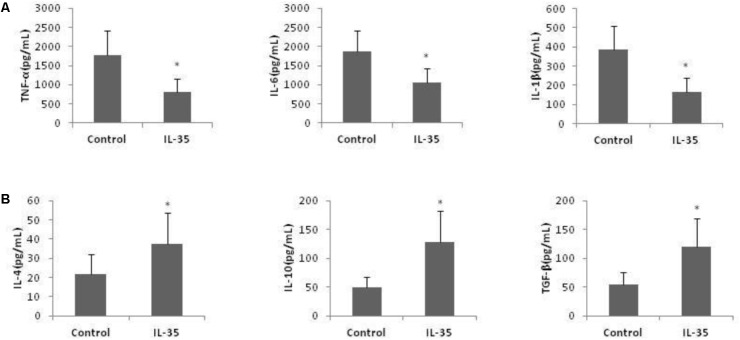
IL-35 affects the cytokines production in endotoxin-induced hepatitis model. **(A)** Pro-inflammatory cytokines TNF-α, IL-1β, and IL-6 production and **(B)** anti-inflammatory cytokines IL-4, IL-10, and TGF-β production were assayed in serum of C57BL/6 mice (*n* = 10) after D-GalN/LPS challenge with or without IL-35 (100 ug/kg). ^∗^*P* < 0.01 vs. control group. Data are representative of three independent experiments.

In addition, the production of pro-inflammatory cytokines, tumor necrosis factor (TNF)-α, IL-1β, and IL-6, was reduced by IL-35 administration, while the production of anti-inflammatory cytokines, IL-4, IL-10, and transforming growth factor (TGF)-β was enhanced (**Figure 2**).

### KCs Were Critical for the Hepato-Protective Role of IL-35

**FIGURE 3:**
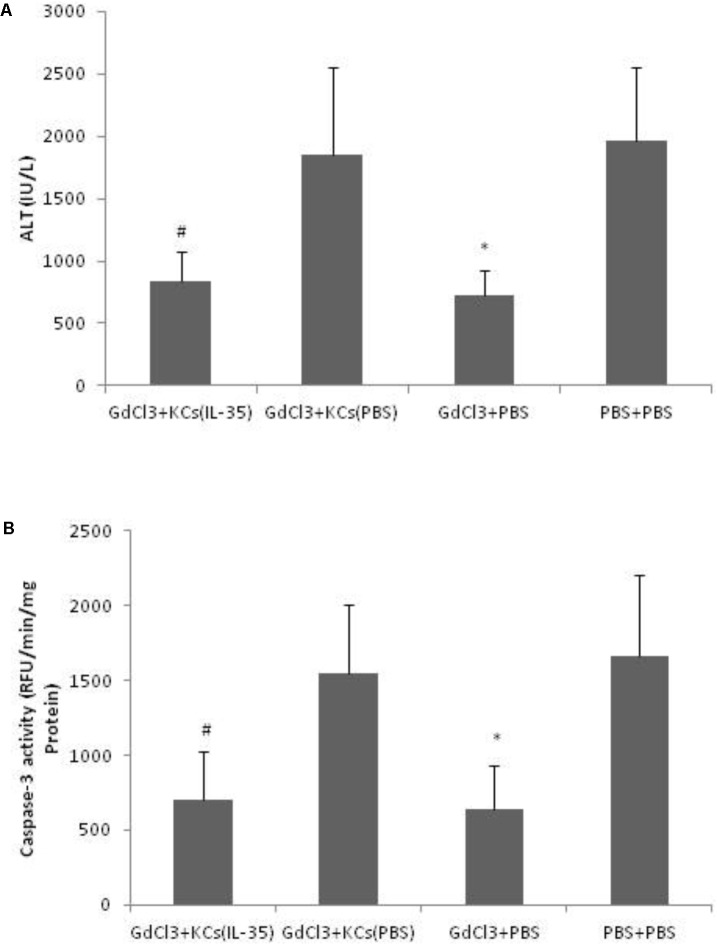
IL-35 protects endotoxin-induced hepatitis via Kupffer cells (KCs). **(A)** Serum ALT and **(B)** Caspase-3 activity were determined in liver tissue of C57BL/6 mice after D-GalN/LPS challenge in different groups (*n* = 8). GdCl3: C57BL/6 mice received GdCl_3_. KCs: p.v. injection of KCs with or without IL-35 (10 ng/ml) pretreatment at 1 day prior to injection. ^∗^*P* < 0.01 vs. PBS + PBS group. ^#^*P* < 0.01 vs. GdCl3 + KCs (PBS) group. Data are representative of three independent experiments.

D-GalN/LPS-induced hepatitis was significantly ameliorated by GdCl_3_, which selectively blocked KCs function. And the hepatic susceptibility to D-GalN/LPS was restored when adoptive transferring of KCs after GdCl_3_ depletion. However, adoptive transfer of KCs, which pretreated with IL-35, significantly protected D-GalN/LPS-induce hepatic injury, suggesting that the hepato-protective effect of IL-35 was mainly mediated by KCs (**Figure 3**).

### *In vitro* Effect of IL-35 on KCs in Response of LPS

**FIGURE 4:**
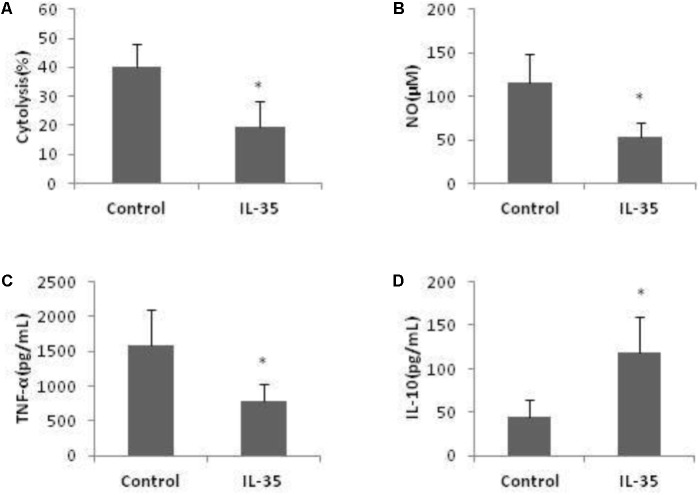
IL-35 reduces cytolysis of LPS-stimulated KCs to hepatocytes through suppression of NO and TNF-α production while induction of IL-10 production. **(A)** KCs from C57BL/6 mice were cultured 3 days prior to stimulation by LPS for 12 h with or without IL-35 (10 ng/ml) (*n* = 8). KCs were then cocultured with hepatocytes at an E: T (KC: hepatocytes) ratio of 1:10. The number of hepatocytes was assayed using anti-F4/80-APC Ab, Annexin-V-FITC, and 7-AAD. The cytolysis (%) = [1-(7AAD^−^Annexin^−^F4/80^−^/F4/80^−^)] × 100. **(B–D)** KCs from C57BL/6 mice were cultured 3 days prior to stimulation by LPS with or without IL-35 (10 ng/ml) (*n* = 8). Supernatant NO, TNF-α, and IL-10 were assayed. ^∗^*P* < 0.01 vs. control group. Data are representative of three independent experiments.

Co-culture of hepatocytes and KCs is a well-known model to investigate liver inflammation. Following LPS activation, KCs exerted potent cytotoxicity against hepatocytes. The number of live hepatocytes was markly reduced when co-cultured with KCs. This cytotoxic effect of KCs was significantly attenuated by IL-35 incubation (**Figure 4A**).

In addition, LPS-induced NO and cytokines production were significantly altered by IL-35 in KCs. Production of NO and TNF-α was significantly inhibited (**Figures 4B,C**), while IL-10 was significantly increased (**Figure 4D**). We did not observe a mark increase of IL-4 and TGF-β by IL-35 treatment. Thus, IL-35 reduced the cytotoxicity of LPS-triggered KCs to hepatocytes and it was related with the suppression of NO and TNF-α production, as well as elevation of IL-10 production.

### Kupffer Cell-Derived IL-10 Was Involved in the Effect of IL-35

**FIGURE 5:**
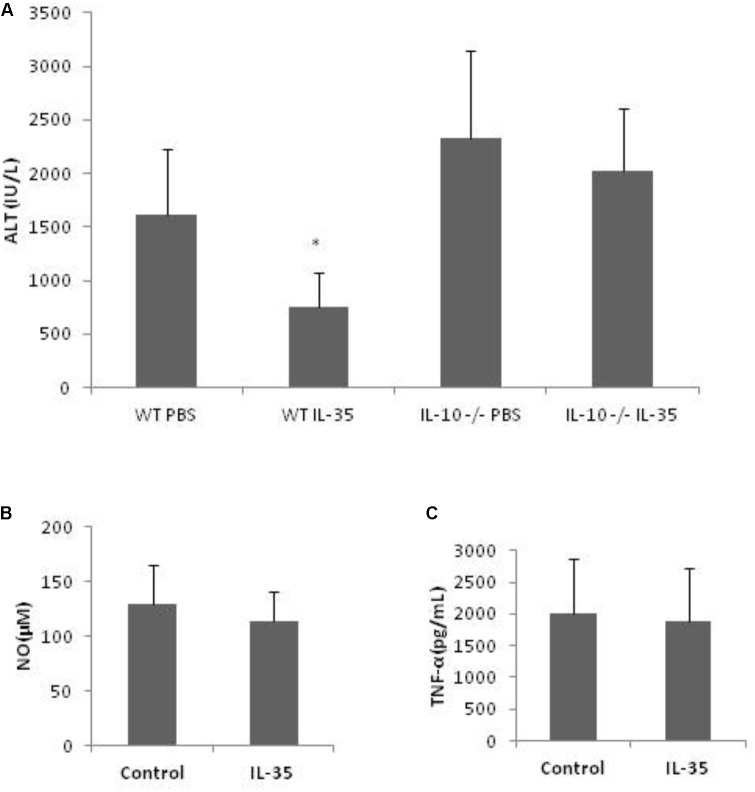
The suppression of endotoxin-induced hepatitis by IL-35 is mediated by KC-derived IL-10. **(A)** Serum ALT determination in IL-10 knockout or WT mice (*n* = 8) after LPS/D-GalN challenge with or without IL-35 (100 ug/kg) *in vivo*. **(B,C)** KCs from IL-10 knockout mice were cultured 3 days prior to stimulation by LPS with or without IL-35 (10 ng/ml) *in vitro* (*n* = 8). Supernatant NO and TNF-α were assayed. ^∗^*P* < 0.01 vs. WT PBS group. Data are representative of three independent experiments.

With the above results, we intended to further know whether IL-10 was critical for KCs’ role in the hepato-protective effect of IL-35, considering the anti-inflammatory property of IL-10. However, neutralizing anti-IL-10 antibody itself could affect D-GalN/LPS inducing hepatic injury (data not shown). Thus, IL-10-deficient mice were used in this experiment. IL-35 could not exert hepato-protective effect in IL-10-deficient mice *in vivo* (**Figure 5A**). Furthermore, the production of NO and TNF-α was not suppressed by IL-35 in IL-10-deficient KCs *in vitro* (**Figures 5B,C**). Therefore, IL-10 derived from KCs was responsible for the protective effect of IL-35 on D-GalN/LPS induced hepatic injury.

## Discussion

Fulminant hepatitis is a devastating syndrome which remains a huge challenge for clinicians. It can be caused by a variety of hepatic insults, such as viruses, drugs, alcohol, autoimmune diseases, and so on. However, endotoxin is considered to play an essential role in the pathological develop of fulminant hepatitis. Using a well-established endotoxin-induced hepatic injury model, this study showed that fulminant hepatitis could be relieved by a potent immunosuppressive cytokine called IL-35. And it was revealed that the hepato-protective effect of IL-35 was mainly medicated by KCs produced IL-10.

D-GalN/LPS induced liver injury is a well-established animal model widely used in exploring the pathogenesis of fulminant hepatitis. For this model, we only used male mice in this research as gender differences were reported in several studies of response to endotoxin treatment (Asai et al., 2001; Coyle et al., 2006; van Eijk et al., 2008). Immune defense capacity often differs between men and women. Generally, whereas men are more prone to infection and sepsis, women more commonly develop autoimmune diseases.

As a new member of IL-12 family cytokine, IL-35 is also a heterodimer protein, which composed of IL-12p35 and EBI3 subunits. Suppression action on inflammation of IL-35 has been observed mostly in autoimmune diseases previously. IL-35 often acts by converting resting T cells into IL-35-producing regulatory T cells (iTR35) and resting B cells IL-35-producing regulatory B cells (IL-35^+^Breg). Recently, more investigators are interested in the anti-inflammatory property of IL-35 in anti-infectious immunity and innate immune system. It was reported that IL-35 was elevated in septic patients (Cao et al., 2015) and IL-35 pretreatment could alleviate LPS-induced acute kidney injury (Jiang et al., 2005). Our previous work revealed that LPS-induced alternatively activated anti-inflammatory macrophages (M2) to classically activated inflammatory macrophages (M1) transformation and IL-12p70 production could be inhibited by Candida albicans, owing to its up-regulation of EBI3 expression (Zheng et al., 2013). Zhang et al found that IL-35 inhibited inflammatory process in Psoriasis, as secretion of inflammatory cytokine and ratio of M1/M2 macrophage were reduced by IL-35, which was consistent with our work (Zhang et al., 2016). Here, we demonstrate that IL-35 supplement played a protective role in endotoxin-induced hepatic injury. Recombinant IL-35 was used as 0.75 ug/day/mouse or 2–10 ug/mouse in previous researches (Niedbala et al., 2007; Singh et al., 2015). To observe the dose effect, IL-35 used in our therapy was 1, 10 and 100 ug/kg BW individually, which was about 0.02, 0.2, and 2 ug for a 20 g mouse. For a maximum effect, dose of 100 ug/kg BW IL-35 was used for the other experiments. In addition, with the help of gadolinium chloride depletion and cell adoptive transfer experiment, we verified that IL-35 protected D-GalN/LPS induced hepatitis mainly mediated by its immunoregulatory functions on KC, a resident macrophage of the liver, as GdCl_3_ could mainly block phagocytosis of KCs (Husztik et al., 1980).

Kupffer cells are the specialized macrophages which only exist in the liver. They play a critical role in host defense and are also associated with various pathophysiologies and toxicities. They have the ability to modulate hepatic inflammation and injury and are also the vital factor accounting for hepatic inflammation and injury. They are the immune cells among the first line exposed and responsible to gut-derived toxins, such as LPS. KCs respond to LPS by secreting pro-inflammatory cytokines (for example, TNF-α, IL-1β, and IL-6) and reactive oxygen species (ROS) (for example, superoxide, and NO) (Wu et al., 2010). By contrast, KCs also produce anti-inflammatory cytokines (for example, IL-4, IL-10, and TGF-β) when stimulated with LPS, which exert suppression effects. In this study, by D-GalN/ LPS induced hepatic injury model, it was shown that IL-35 not only reduced the production of pro-inflammatory cytokines (TNF-α, IL-1β, and IL-6), but also elevated the production of anti-inflammatory cytokines (IL-4, IL-10, and TGF-β). KCs-derived IL-10 accounted immune tolerance to LPS with significant physiological and pathophysiological meaning (Guo and Friedman, 2010). By using of IL-10-deficient mice, we found KCs-derived IL-10 played vital role in the hepato-protective effect of IL-35. IL-35 enhanced IL-10 secretion in KCs, which suppressed pro-inflammatory cytokines and ROS production and eventually alleviated the liver damage. IL-4 was considered as a Th2 cytokine and also contributed to alternative activation of macrophages (M2 polarization), which downregulated inflammatory mediators (Lopez-Navarrete et al., 2011). IL-4 ameliorated BCG/LPS-induced liver injury, and inhibited TNF-α, IL-1β, IFN-γ, IL-6, and iNOS mRNA in liver (Zhang et al., 2012). Although being a pro-inflammatory cytokine, IL-6 could be a protective factor in immune resistance to LPS in macrophages (Menghini et al., 2014). It was reported that IL-6 infusion attenuated endotoxin-induced TNF-α production in humans (Starkie et al., 2003). And IL-6 protected against LPS/D-Gal-induced acute liver injury and inhibited inflammatory responses in KCs (Li et al., 2017). However, IL-35 treatment reduced hepatic IL-6 production, which meant that the hepato-protective effect of IL-35 was not related with IL-6. Furthermore, the experiments on IL-10 deficient mice revealed that the hepato-protective effect of IL-35 was mainly mediated by IL-10, neither IL-4 nor IL-6.

Although the receptor of IL-35 has been identified, the signaling transduction pathway of IL-35 is complicated and is still not well-understood. And the two subunits of IL-35, EBI3, and IL-12p35, also have their own function (Dambuza et al., 2017). Accordingly, to find the therapeutic target of treating hepatitis, the molecular mechanism of IL-35 affecting KCs-derived IL-10 needs to be studied in the future.

## Conclusion

This paper illustrates that IL-35, an immunosuppressive cytokine of IL-12 family, plays a protective effect against endotoxin-induced hepatitis. The augmentation of KCs produced IL-10 by IL-35 might be responsible for this effect. We get a further understanding of the target cell and the biological role of IL-35 in anti-infectious immunity. This study is also expected to provide evidence for finding therapeutic target in the treatment of fulminant hepatitis.

## Author Contributions

X-FZ, BM, S-CX, and Z-FX conceived and designed the experiments. X-FZ, X-YH, HF, and FZ performed the experiments. X-FZ, Y-FM, and F-YY analyzed the data. S-CX and Z-FX contributed reagents, materials, and analysis tools. X-FZ, BM, and Y-FM wrote the paper.

## Conflict of Interest Statement

The authors declare that the research was conducted in the absence of any commercial or financial relationships that could be construed as a potential conflict of interest.The reviewers CF and GO and handling Editor declared their shared affiliation.
